# Brain-Derived Neurotrophic Factor (BDNF) as an Indicator for Effects of Cognitive Behavioral Therapy (CBT): A Systematic Review

**DOI:** 10.3390/biomedicines11010027

**Published:** 2022-12-22

**Authors:** Anna Mosiołek, Magdalena Pietrzak, Maria Tabisz, Wiktoria Wojtaszek, Michalina Zabielska, Agnieszka Ostrowska, Paweł Szwed, Jadwiga Mosiołek, Agata Szulc

**Affiliations:** 1 Faculty of Health Sciences, Medical University of Warsaw, 02-091 Warsaw, Poland; 2 Institute of Psychology, Cardinal Stefan Wyszynski University, 01-938 Warsaw, Poland; 3 Faculty of Medicine, Wroclaw Medical University, 50-367 Wrocław, Poland

**Keywords:** brain-derived neurotrophic factor (BDNF), psychotherapy, cognitive-behavioral therapy (CBT)

## Abstract

Brain-derived neurotrophic factor (BDNF) is a protein affecting survival of existing neurons and neuronal maturation. Patients suffering from several mental disorders exhibit reduced BDNF levels comparing to healthy population. In this systematic review we aim to evaluate the effect of broadly defined cognitive behavioral therapy (CBT) on BDNF levels in psychiatric patients. A literature search was performed using PubMed and Google Scholar data bases. The resources were searched between 14 January and 3 February 2022. Following the inclusion criteria, a total of 10 randomized-controlled trials were included. The results of our research indicate that BDNF levels might be considered an indicator of a result achieved in psychotherapy of cognitive functions. However, no such correlation was observed for mindfulness-based practices intended to lower stress levels or improve the quality of life. It is important to notice that present research showed no consistent correlation between the increase in BDNF levels and the perceived effectiveness of the procedures. Thus, the exact role of BDNF remains unknown, and so far, it cannot be taken as an objective measure of the quality of the interventions.

## 1. Introduction

Brain-derived neurotrophic factor (BDNF) is a growth factor synthesized in the cell bodies of neurons and glia. It affects neuronal maturation [1], survival of neurons in the nervous system [2], and synaptic plasticity [3]. 

BDNF is concentrated in certain regions of the brain including the prefrontal cortex and the hippocampus [4]—regions where complex cognitive processes such as memory, personality, and emotional control occur. BDNF is released during acute stress response, mainly in the limbic system [5]. Patients suffering from psychiatric disorders exhibit lower levels of BDNF than healthy controls [6,7,8]. BDNF has been proposed as a putative candidate biomarker for mood disorders [9] and schizophrenia [10].

Due to the fact that psychotherapy stimulates the above-mentioned areas [11], researchers wonder whether psychotherapy can cause an increase in the level of BDNF. There are a few clinical studies concerning the change in BDNF levels following psychotherapy. The authors collected them according to the inclusion criteria and present the data in the present review. In this study, we evaluate possible changes in BDNF levels in people, both healthy controls and patients suffering from mental disorders, such as schizophrenia, cognitive impairment of various causes, depression, and obsessive-compulsive disorder (OCD), following psychotherapy. This review includes: Cognitive Training, Cognitive Behavioral Therapy (CBT), Neurofeedback, and Mindfulness. 

## 2. Materials and Methods

**Figure 1:**
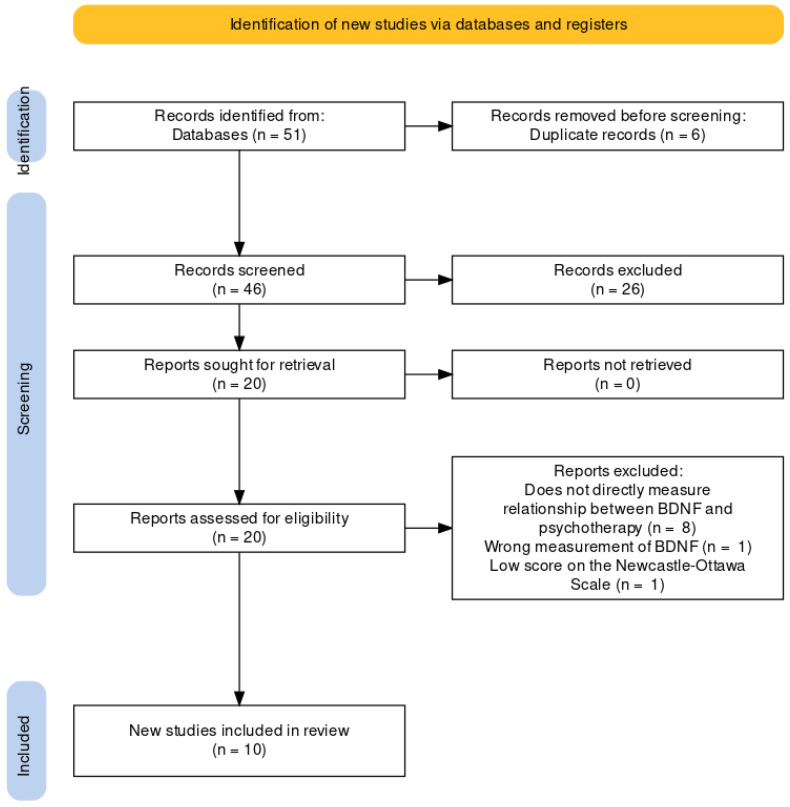
PRISMA flow diagram for the systematic review.

This review adheres to the Preferred Reporting Items for Systematic Reviews and Meta-Analyses [12]. The protocol was registered on PROSPERO: CRD42018108144 (Figure 1). 

The titles were obtained by nine independent authors by searching the following databases: PubMed, Google Scholar, and accessed through EBSCOhost: Contemporary Index, Medicine Complete, Academic Search Ultimate, Medline, Springer Nature Journals, Scopus, Health Source: Nursing/Academic Edition, APA PsychInfo, ScienceDirect, APA PsycArticles. One more study was identified using ConnectedPapers. The resources were searched from 14 January to 3 February 2022. Only articles written in English and after 2008 were considered.

The search terms included: BDNF and psychotherapy. Cognitive behavioral therapy (CBT) was not included in search terms; however, most results were related to it, so non-CBT studies were removed later on. 

The inclusion criteria were organized according to the PICO framework. The population that we were interested in was adults with mental health issues or cognitive impairment. As for the intervention, only studies that used some forms of CBT (such as Cognitive Training, Mindfulness-based programs, individual or group CBT) were included. To compare studies, we required them to have a form of a randomized-controlled trial and a control group that would allow to isolate the effect of psychotherapy. To determine if there is a change in the levels of BDNF in serum we also required the studies to mention their levels before and after interventions. The last element of the framework is outcome. The only measure of concern was a change between the BDNF serum levels.

The records were selected by nine independent reviewers. Each one was assessed by one of them. All disagreements were discussed among the authors until a consensus was obtained. Records were first identified by their title, then screened based on their abstract and, later on, the full text. Then one reviewer scanned all qualified records again. No automation tools were used in the process. 

One instrument was used to evaluate the risk of bias in selected records: The Newcastle-Ottawa [13]. One study was removed from the review based on it. Detailed information is presented in Table 1.

## 3. Results

The review focused on impact of broadly defined cognitive-behavioral therapy, such as cognitive training, mindfulness-based programs, and individual or group CBT. The procedures had been conducted on patients with different mental health issues such as schizophrenia, cognitive impairment of various causes, depression, and obsessive-compulsive disorder (OCD). Detailed information on reviewed papers is summarized in Table 2.

Vinogradov et al. [14] conducted a 10-week study on schizophrenic patients taking part in computer-based cognitive training. The study generally included 72 subjects, who were assigned to three comparison groups. From a sample of 56 clinically stable, chronically ill, outpatient patients, 30 subjects were randomly assigned to 50 h of cognitive training and 26 to computer games as a placebo. Targeted cognitive training of auditory and verbal processing was performed using software developed by Posit Science Corporation. In these exercises, subjects were tasked with making increasingly accurate distinctions about the spectrotemporal structure of fine auditory and speech stimuli under increasing working memory load and to incorporate and generalize these improvements to language comprehension. The study also included a 16-person control group of healthy subjects matched for age, gender, body mass index (BMI), smoking history, and education. All groups were compared in terms of baseline cognitive scores and serum BDNF. The main goal was to improve the patients’ auditory process while observing whether such training could result in increased serum BDNF levels. Serum BDNF levels measured at baseline were significantly lower in the test group than in the control group. Blood samples were taken from all participants 2–3 weeks after the start of the study, while in those with schizophrenia, blood was drawn an additional two times: after 10 h of cognitive training (2 weeks) and again after completing 50 h of interaction (10 weeks). After 2 weeks, an increase in BDNF levels was observed in the treated patients. On the other hand, after cognitive training, the level of neurotrophic factor reached an average serum level comparable to the healthy population. Despite the increased BDNF levels in the cognitive training group, there was no relationship between the change in BDNF and the improvement in cognitive function. The increase in the concentration of neurotrophic factors, on the other hand, correlated with improvements in quality of life. In the control group, no changes in BDNF levels were observed throughout the study. 

Similar results were obtained by Fisher et al. [15] in a study of computer-based cognitive training on 88 patients with schizophrenia. The subjects were randomly distributed to either targeted auditory training or computer games constituting the control group. Serum BDNF levels were assessed at baseline after 2 weeks and after 10 weeks of training. Patients in the study group showed a significant increase in serum BDNF levels after training compared to participants in the control group and made significant cognitive gains. However, again in this study, the increase in BDNF was not correlated with cognitive improvement.

Jeong et al. [16] also published a study aiming to enhance cognitive functions. The sample consisted of 293 amnestic patients with mild impairment in this area. More and more people are suffering from Amnestic Mild Cognitive Impairment (aMCI), which often is a precursor to dementia. Unfortunately, pharmacology is not used in the treatment of aMCI and it is not known how to treat this disorder. So, studies concerning the effects of cognitive training on aMCI are very needed. Jeong’s study was conducted in 18 neurology clinics of nationwide hospitals in South Korea. Subjects were randomly assigned to three groups: group-based cognitive intervention (GCI), home-based cognitive intervention (HCI), and control group. The first group (GCI) participated in 90-min training sessions, twice a week and the second group (HCI) completed homework materials consisting of seven pages, 5 days per week. Subjects from the second group visited a clinic every week in the 1st month and every other week in the 2nd and 3rd months. Health professionals controlled and checked their homework. Training program (GCI and HCI) was conducted by previously trained specialists (e.g., psychologists and therapists). Serum BDNF levels in patients undergoing group-based (GCI) and home-based cognitive intervention (HCI) increased significantly after the 12-week participation. Jeong’s project indicated the existence of a correlation between BDNF increase and the improvement of cognitive functions. 

Different results were obtained by Penades et al. [17]. In their 4-month intervention, patients with a diagnosis of schizophrenia and resulting cognitive impairment were subjected to cognitive training. The procedure included exercises aimed at improving cognitive flexibility, working memory, information processing, and planning. BDNF serum levels were measured before the start of the interactions, after 4 weeks of treatment, and at the end of the procedure. However, no significant differences were observed between the results obtained in any of the measurements, despite the observed improvements in cognitive ability and quality of life, as measured psychometrically. Moreover, initial BDNF levels did not differ between the clinical trial and the healthy control sample. Interestingly, controlling for the genetic variant of BDNF, differences were seen in Val/Val variant carriers, but not among Val/Met variant carriers. This suggests that the genotype of Met carriers could be acting as a marker of a negative response. The authors also suggest that the lack of a significant result may be due to the method of BDNF measurement. In the current study, serum levels were measured, while promising results based on plasma BDNF measurement can be found in the literature. The genetic basis and the method of measuring BDNF levels are possible directions for further research.

Another study, based on mindfulness practice, was conducted by Nery et al. [18]. The subjects were women struggling with infertility, and the resulting stress and depressive symptoms. As part of the interactions, the researchers applied MBP practice, focused on promoting self-awareness, self-reflection, and self-care. The procedure has a proven track record of improving the perceived quality of life. BDNF has been identified as a potential objective indicator of MBP’s effect since in healthy women its levels correlate with resilience and stress coping. In addition to measuring BDNF levels, cortisol levels in a hair sample were also examined. Each subject participated in a weekly two-hour MBP practice over a 10-week period. One session consisted of meditation practice, relaxation, autogenic training, guided imagery, and biofeedback. Measurements were taken one week before the interactions began and one week after the interactions ended. Despite the perceived reduction in symptoms as measured by questionnaires, the increase in serum BDNF levels was insignificant. As a possible reason for the lack of reflection of the effects of the therapeutic interaction on BDNF levels, the authors point to the timing of the measurement, which may be inadequate in relation to when the effect occurs. More research is needed, to explore the appropriate timing of BDNF level measurement.

The effectiveness of cognitive training was also measured in healthy elders by Ledreux et al. [19]. One hundred and forty-six healthy older individuals from USA and Sweden participated in the study. Subjects were randomly divided into the following four conditions: physical exercise, cognitive training, mindfulness practice, or control condition. All interventions were structurally similar, using interactive, computer-based software. The subjects participated in one of these activities for 35 min a day, 5 days per week, for 5 weeks. After a five-week computer-assisted procedure consisting of various cognitive tasks, serum BDNF levels in the subjects turned out to be significantly higher than those before the study. Ledreux’s study also included a mindfulness-based procedure conducted on a parallel sample of healthy elders; however, it did not increase the level of BDNF. The physical exercise also did not cause a significant change in BDNF levels after 5 weeks of training. Consequently, it can be assumed that only cognitive training is a procedure that can induce improvements in the elderly reflected in an increase in BDNF levels. However, further research is needed on the specifics of the effectiveness of interventions and the associated increase in BDNF levels.

In the study conducted by Bruijniks et al. [20], patients with major depressive disorder underwent therapeutic interventions of cognitive-behavioral psychotherapy and IPT. However, no significant changes were observed in serum BDNF levels associated with differences in session frequency (once versus twice a week) or treatment procedures (CBT versus IPT). There were also no changes in BDNF levels in the results after 6 months. The results of this study may suggest that BDNF levels do not have a role in the effects of psychotherapy in the treatment of depression, which contrasts with the results of other studies. The authors consider the impact of small sample size and population dependence (differences between disorders). Nonetheless, the study shows the contribution of working memory to the relationship between baseline BDNF and outcomes. In patients with high working memory, higher baseline BDNF was associated with lower levels of depression after treatment. This overlaps with the results of previous works where a relationship was found between working memory capacity and symptom change. This may indicate that there is a subgroup among patients with depression where working memory and BDNF levels are predictors of treatment processes.

Another study was conducted by Viol et al. [21] on patients suffering from obsessive-compulsive disorder. In the study consisting of 17 patients diagnosed using ICD-10 and DSM-IV criteria. Participants were tested before and after inpatient psychotherapy. The tests were used to determine symptom severity and BDNF level among other measures. Three different scales were used to determine the severity of the symptoms: Global Severity Index of the Symptom Checklist-90-R for overall symptom severity, Beck Depression Inventory II for depressive symptoms, Yale-Brown Obsessive-Compulsive Scale for obsessive-compulsive symptom severity. The study concluded that participation in individual psychotherapy sessions significantly reduces patients’ symptoms and increases BDNF levels. Though, the last difference was not statistically significant.

Liou et al. [22] investigated the relationship between BDNF level in serum and treatment type (cognitive-behavioral therapy for insomnia, CBT-I; and acupuncture). All interventions lasted for eight weeks. The sample consisted of 160 cancer survivors with insomnia. Participants were not limited by their cancer type or stage, as long as they had been receiving active cancer treatment for at least one month prior to the study’s start. The severity of symptoms was measured using Insomnia Severity Index (ISI) and a consensus sleep diary (CSD). The ISI is a measure that was reliably used before in populations affected by cancer [24] and CSD provides a standardized way to measure sleep time and quality [25], in the study only total sleep time was included in the analyses. To qualify for study, one needed a score of at least 8 on the ISI as well as meeting the criteria for insomnia according to the DSM-5. According to the findings, patients who underwent CBT-I had significantly lower ISI scores and insignificantly higher BDNF levels. 

Another trial on schizophrenia patients was conducted by Markiewicz et al. [23]. The sample contained 44 participants currently in stable, incomplete disease remission (without active psychotic symptoms for at least 18 months). The subjects were restricted to male patients to eliminate the influence of sex differences such as phases of the menstrual cycle, which can affect the level of BDNF [26]. Subjects were randomized to two groups, with standard rehabilitation and the use of neurofeedback (NF) as an additional procedure supporting the standard rehabilitation. The standard rehabilitation program consisted of continued treatment as usual in a day care center (psychopharmacotherapy, clinical management, and standard daily care). The study lasted 3 months and during this time NF training was held twice a week. The increase in BDNF levels was significantly higher in the NF patients than in controls undergoing only the usual form of rehabilitation. The result of this study suggests that neurofeedback training may strengthen the cognitive, clinical, and psychosocial rehabilitation of patients with schizophrenia, resulting in the increase in serum BDNF levels.

## 4. Discussion

This paper reviews the literature on the role of BDNF, a neuronal and glial cell growth factor, in validating the efficacy of therapeutic interventions within the broad framework of CBT. Although BDNF has been cited as a possible objective indicator of change noted as a result of therapeutic interventions, the evidence so far has not been clear.

According to the research included in our review, the change in BDNF levels might be considered an indicator of a result achieved in psychotherapeutic procedures aiming to improve cognitive functions [14,15,16,19,23]. However, it does not seem to be applicable for the treatment intended to lower stress levels or improve the quality of life, such as mindfulness-based practices [18,19,21,23]. Whether the results of psychotherapy focusing mainly on cognitive methods aiming to alleviate the symptoms of mental health issues and improve the quality of life are also reflected in the changes in BDNF levels remains unclear. This issue seems to be worth considering in subsequent studies.

It is essential to note that the presented research did not suggest a consistent correlation between the increase in BDNF levels and the perceived effectiveness of the procedures [14,15,17,18]. Thus, the exact role of BDNF remains unknown, and so far, it cannot be taken as an objective measure of the quality of the interventions. There is a possibility that the change depends on the initial level of BDNF, but more precise data on the results obtained in individual measurements are needed to prove this thesis. Changes in BDNF may also depend on the type of symptoms experienced and how the improvement was determined.

Additionally, the results presented here highlight two issues worthy of close examination. The first of these concerns the role of baseline BDNF levels in the changes in its amount following therapeutic interventions. As mentioned above, in studies on the effectiveness of cognitive training in a group of people with schizophrenia, the level of BDNF was not in every case significantly different from that recorded in the healthy population [14,15,17]. Since the results of the described studies also varied, it seems to be worth examining this issue in more detail. Secondly, studies indicate differential responses to treatment depending on the genetic variant of BDNF, which should also be considered in future work on this topic [17].

A variable that has received little attention in most papers is how BDNF levels are measured. Serum BDNF levels were conducted in each of the included studies. However, the data included in the systematic review conducted by Tikhonova et al. [27] suggest that plasma or serum levels do not correlate with brain BDNF levels. More studies including the measures in the central nervous system are needed.

Finally, it needs to be mentioned that research on BDNF as an indicator of the effectiveness of psychotherapeutic interventions is limited and conducted on small samples. In order to draw certain conclusions on this subject, a deeper study is required.

## 5. Conclusions

Despite the many promising past results referred to by the researchers, the above review indicates that, at present, BDNF levels cannot function as an objective indicator of the change observed as a result of psychotherapeutic interventions, and therefore cannot provide evidence of the effectiveness of psychotherapy. More research is needed, addressing the issues mentioned in the discussion in more detail. A thorough investigation of the relationship between initial BDNF levels and observed improvement is needed. The genetic variant of the protein needs to be controlled. More research needs to clarify doubts about such topics as the optimal timing of measurement or the relationship between change and the type of interventions. Finally, attention also needs to be given to how BDNF levels are measured, as the usual measurement of peripheral levels may not be adequate. The current state of research shows that we are only at the beginning of the road to considering the role of BDNF in the assessment and planning of psychotherapeutic interventions. Nevertheless, as the results obtained in some cases show, this is still a promising and worthwhile area of research. 

## Figures and Tables

**Table 1:** Evaluation of risk bias.

Study	Selection				Comparability	Outcome	
	Case Definition	Representativeness of the Cases	Selection of Controls	Definition of Controls		Ascertainment of Exposure	Non-Response Rate
Vinogradov et al., 2009 [14]	*		*		**	*	
Fisher et al., 2016 [15]	*		*		*	*	*
Jeong et al., 2016 [16]	*	*	*		*	*	*
Penades et al., 2017 [17]	*		*		**	*	*
Nery et al., 2018 [18]	*		*		*	*	
Ledreux et al., 2019 [19]	*		*		**	*	
Bruijniks et al., 2020 [20]	*					*	
Viol et al., 2020 [21]	*					*	
Liou et al., 2021 [22]	*		*	*		*	
Markiewicz et al., 2021 [23]			*		*	*	

* represent the quality of choices made at each stage of the research (0 stars being the worst, and ** being the best).

**Table 2:** Summary of the papers reviewed.

Study	Year of Study	Patient Group	Duration of Study (Weeks)	Disorder	Therapy	Results	Effect on BDNF (- no Effect, + Effect Observed)
Vinogradov et al. [14]	2009	n = 72	10	Schizophrenia	Computerized auditory training	The BDNF level increased after 2 weeks of training and matched the level in healthy adults after 10 weeks. Control group-no effect. No correlation between BDNF increase and improved cognition immediately after the intervention.	+
Fisher et al. [15]	2016	n = 88	10	Schizophrenia	Computerized cognitive training	Significant increase in BDNF levels during the procedure and levels similar to healthy adults after completed training; however not connected to the cognitive improvement.	+
Jeong et al. [16]	2016	n = 293	12	Amnestic mild cognitive impairment	Group-based cognitive intervention, home-based cognitive intervention	The level of serum BDNF in patients undergoing the procedures increased significantly. The BDNF level correlated with improvement of cognitive functions.	+
Penades et al. [17]	2017	n = 70	4 months	Schizophrenia	Cognitive remediation treatment	No significant differences between the CRT group and controls, no significant differences from the pre-treatment BDNF levels, despite improvements in cognitive functions and quality of life; different responses depending on BDNF genetic variants (however, the effects are still insignificant).	-
Nery et al. [18]	2018	n = 99	8	Stress in infertile women	Mindfulness-based program	The BDNF levels did not change significantly neither in the control group nor in the group undergoing the intervention, although the perceived stress and quality of life improved.	-
Ledreux et al. [19]	2019	n = 146	5	Cognitive impairment in healthy elders	Cognitive training/mindfulness practice	Cognitive training significantly increased BDNF levels, no effect of mindfulness training on BDNF levels.	+/-
Bruijniks et al. [20]	2020	n = 138	16–24	Major Depressive Disorder (MDD)	CBT	No significant change in BDNF levels post-treatment.	-
Viol et al. [21]	2020	n = 34	86 days	Obsessive-compulsive disorder (OCD)	CBT, mentalization/mindfulness training, DBT	The BDNF levels increased, but the change was non-significant.	-
Liou et al. [22]	2021	n = 160	8	Insomnia in cancer survivors	CBT	The BDNF did not increase significantly even after controlling the baseline levels.	-
Markiewicz et al. [23]	2021	n = 44	3 months	Schizophrenia	Neurofeedback (NF)	The BDNF levels increased significantly in patients after the NF procedure.	+

## Data Availability

Not applicable.
